# PI and T9-SPI: New Predictive Factors for Increased Kyphosis of the Thoracolumbar Junction in Thoracolumbar/Lumbar Adolescent Idiopathic Scoliosis

**DOI:** 10.3389/fped.2020.520086

**Published:** 2020-11-12

**Authors:** XiaoLong Lin, Jie Zhu, Weiping Sha, Fei Yan, Liming Wang, Yong Qiu

**Affiliations:** ^1^Department of Orthopaedic Surgery, The Affiliated Zhangjiagang Hospital of Soochow University, Zhangjiagang, China; ^2^Department of Anesthesiology, The Affiliated Zhangjiagang Hospital of Soochow University, Zhangjiagang, China; ^3^Department of Spine Surgery, Affiliated Drum Tower Hospital of Nanjing University, Nanjing, China

**Keywords:** adolescent idiopathic scoliosis, thoracolumbar junction, pelvic incidence, T9-SPI, kyphosis

## Abstract

**Objectives:** Studies have demonstrated that there is an increased thoracolumbar junction sagittal Cobb angle (TLJS) in thoracolumbar/lumbar adolescent idiopathic scoliosis (AIS) patients. The objectives were to ascertain the correlations between the spinopelvic alignments and TLJS and to explore potential predictive factors for hyperkyphotic TLJS in the sagittal plane in thoracolumbar/lumbar AIS.

**Methods:** A total of 114 AIS patients with thoracolumbar/lumbar curve were included. Cobb angle, apical vertebrae rotation (AVR), thoracic kyphosis (TK), TLJS, lumbar lordosis (LL), pelvic incidence (PI), sacral slope (SS), pelvic tilt (PT), T1-spinopelvic inclination (T1-SPI), and T9-spinopelvic inclination (T9-SPI) were measured. After patients were organized into two subgroups based on TLJS, all parameters were compared between the two groups. Correlation analysis and multiple linear regression analysis were performed between the radiologic measurements and TLJS in all patients.

**Results:** There was a significant difference between the non-kyphotic group and kyphotic group in mean Nash-Moe grade, TK, T9-SPI, PI, and SS. Correlation analysis showed that LL, PI, and SS were inversely associated with TLJS. TK, T9-SPI, and Nash-Moe grade were positively related to TLJS. The multiple linear regression analysis showed that TLJS could be predicted by the equation TLJS = −2.322 + 5.585 × Nash-Moe grade + 0.687 × T9-SPI – 0.208 × PI, with an adjusted R^2^ of 0.410.

**Conclusion:** TLJS was positively correlated with greater AVR in the coronal plane, greater T9-SPI in the sagittal plane and inversely associated with PI among patients with thoracolumbar/lumbar scoliosis. Spine surgeons should pay more attention to the degree of AVR, T9-SPI, and PI when dealing with thoracolumbar/lumbar scoliosis with thoracolumbar junction kyphosis.

## Introduction

Adolescent idiopathic scoliosis (AIS) is a common and complex three-dimensional deformity of the spine involving the interaction of lateral deviation in the coronal plane, modifications of the sagittal profile and vertebral rotation in the transverse plane of the spine (1). Sagittal spinal alignment and pelvic morphology may both contribute to the development and progression of AIS (2–4). Scoliosis progresses faster in patients with minor thoracic kyphosis (5). However, neither thoracic hypokyphosis nor lumbar hypolordosis is considered to be the initial determinant factor of scoliosis but leads to the progression of scoliosis (5, 6). The pelvic vertebra, as called by Dubousset (7), has been proven to influence the shape and balance of the spine along the sagittal plane, which is specific and constant for each individual. Pelvic incidence (PI), a representative parameter, was significantly greater in AIS patients than in normal adolescents without scoliosis (8, 9). However, the PI in AIS patients was similar to that of normal adolescents according to Yong's study (10) and Ma's study (11).

Van Loon et al. (12) recently demonstrated that a slight or moderate kyphotic thoracolumbar junction has been observed in adolescents with idiopathic scoliosis with a double major curve pattern, i.e., one located between the lower thoracic vertebra and the upper lumbar vertebra of the spine. Furthermore, correction of curves in double curve scoliosis can benefit from a lordotic fulcrum force in the sagittal plane on the thoracolumbar junction region (12). In addition, an increased thoracolumbar junction sagittal Cobb angle (TLJS) has also been identified in thoracolumbar/lumbar AIS (8, 13). It has also been mentioned that hyperkyphotic TLJS plays an important role in the development of symptomatic sagittal imbalance (14).

TLJS has been demonstrated to be positively correlated with greater axial vertebral rotation of the scoliotic spine in the coronal plane (13). As no study examining the correlation between sagittal spinopelvic morphology and the magnitude of TLJS in AIS patients has been published, we analyzed a series of thoracolumbar/lumbar AIS patients with or without kyphotic TLJS to ascertain the correlation, if any, between spinopelvic alignment and TLJS. More importantly, we explored the potential predictive factors for hyperkyphotic TLJS in the sagittal plane.

## Materials and Methods

### Patients

**Figure 1:**
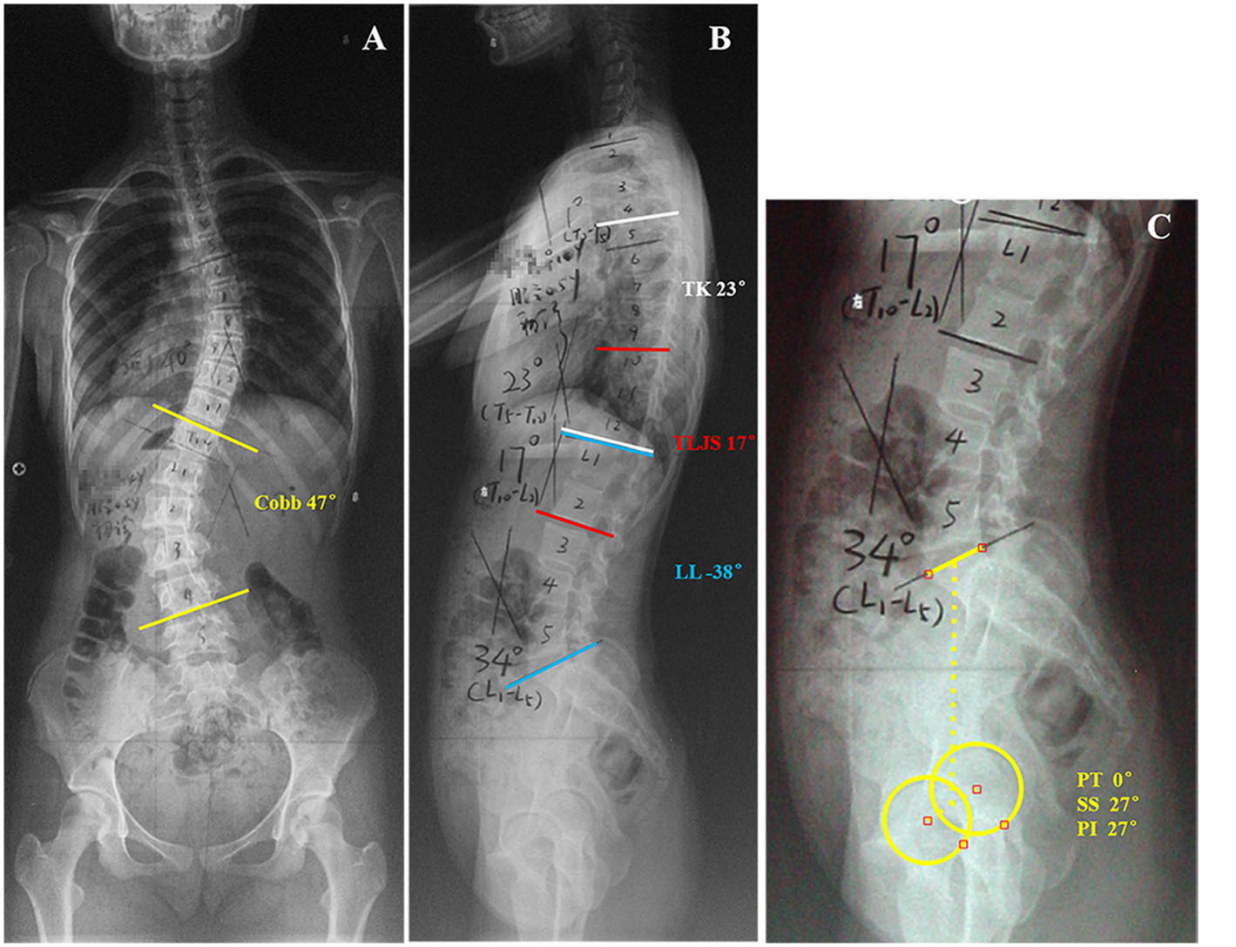
This is a 14-year-old girl with Lenke type 5 adolescent idiopathic scoliosis **(A)** Preoperative anteroposterior radiograph idiopathic scoliosis showed 47° primary thoracolumbar/lumbar curve (T12-L4). **(B,C)** Preoperative lateral radiograph of the same patient showed: thoracic kyphosis (TK), 23°; lumber lordosis (LL), −38°; 17° kyphosis of the thoracolumbar junction sagittal Cobb angle (TLJS); sacral slope (SS), 27°; pelvic tilt (PT), 0°; pelvic incidence (PI), 27°.

**Figure 2:**
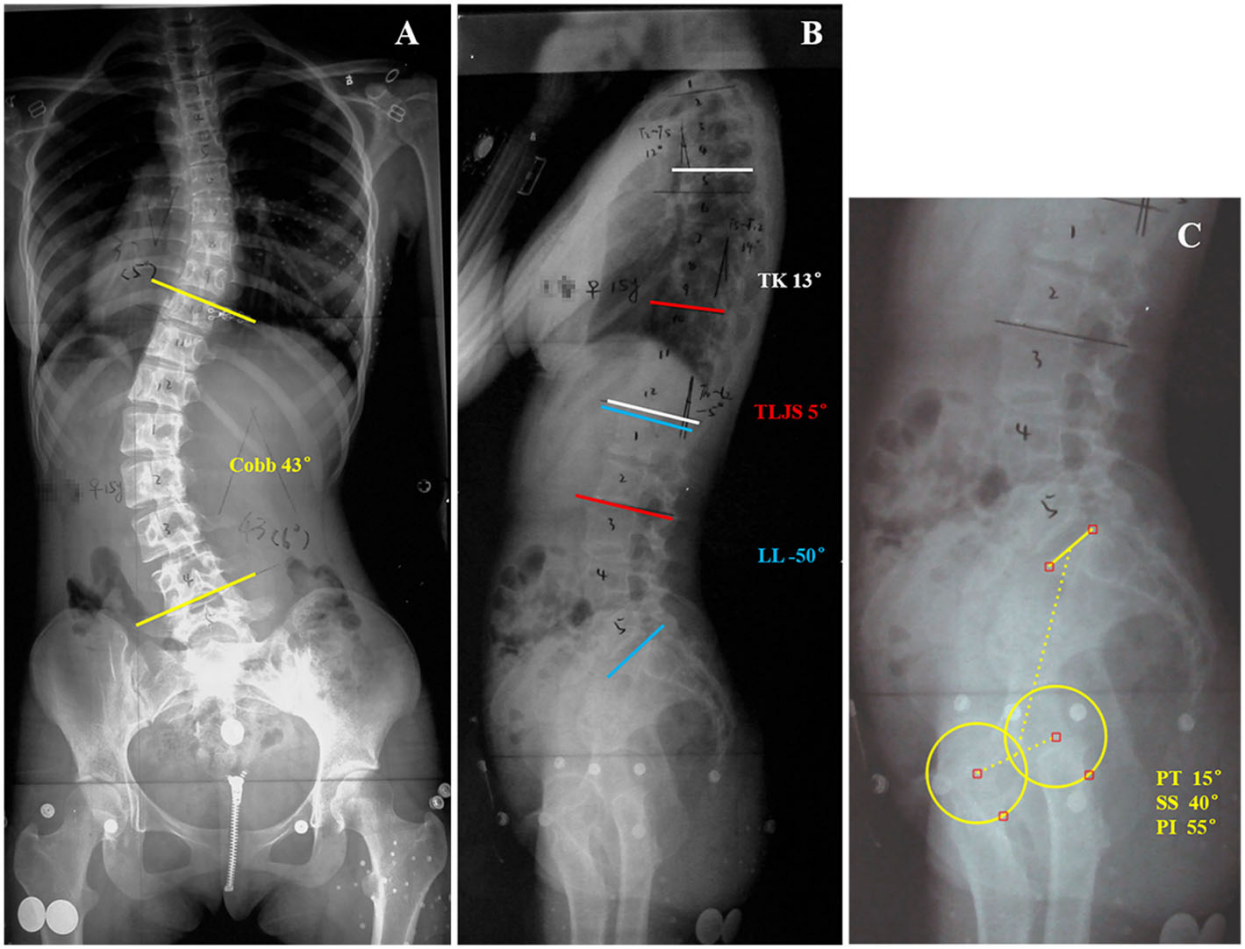
This is a 15-year-old girl with Lenke type 5 adolescent idiopathic scoliosis **(A)** Preoperative anteroposterior radiograph idiopathic scoliosis showed 43° primary thoracolumbar/lumbar curve (T10-L4). **(B,C)** Preoperative lateral radiograph of the same patient showed: thoracic kyphosis (TK), 13°; lumber lordosis (LL), −50°; 5° kyphosis of the thoracolumbar junction sagittal Cobb angle (TLJS); sacral slope (SS), 40°; pelvic tilt (PT), 15°; pelvic incidence (PI), 55°.

This study was approved by the ethics review board of the Affiliated Zhangjiagang Hospital of Soochow University, and the methods were carried out in accordance with the guidelines and details from the ethics review board. A total of 114 thoracolumbar/lumbar AIS patients were included in the current study. The diagnosis of thoracolumbar/lumbar AIS was confirmed by comprehensive physical examination by senior spine doctors and standard radiological examination after excluding other possible causes of scoliosis according to the Lenke classification system of AIS (15). None of these patients had undergone any previous treatment for scoliosis. The AIS patients were organized into two subgroups based on TLJS, which has been considered physiological if TLJS ≤ +10° (16): the kyphotic group included patients with TLJS > +10° (Figure 1), and the non-kyphotic group included patients with TLJS ≤ +10° (Figure 2). Informed consent was obtained from all subjects or their parents.

### Radiographic Evaluation

Standard long-cassette standing upright anteroposterior (AP) and lateral radiographs of the spine and pelvis were obtained from each patient. The AP radiograph of each patient was measured regarding the following: coronal Cobb angles of the primary thoracolumbar/lumbar curve, location of the apex (vertebra or disc) of the primary thoracolumbar/lumbar curve, and degree of apical vertebrae rotation (AVR), which is commonly estimated by the Nash-Moe method using plain AP radiographs of the spinal column. If the apex was a disc, then the AVR was calculated by the average of the grades of the 2 adjacent vertebrae that were above and below the disc.

Radiographic parameters of alignment of the 3 pelvic and 5 spinal parameters in the sagittal plane were measured on each lateral radiograph:

1. Pelvic incidence (PI; the angle between the perpendicular to the sacral plate midpoint and the axis of the femoral heads).
2. Sacral slope (SS; the angle between the horizontal line and the sacral plate).
3. Pelvic tilt (PT; the angle between the vertical plane and the line connecting the sacral midpoint to the femoral heads).
4. Thoracic kyphosis (TK; the angle between the upper endplate of the T5 vertebra and the lower endplate of the T12 vertebra).
5. Lumbar lordosis (LL; the angle between the upper endplate of the L1 vertebra and the upper endplate of the S1 vertebra).
6. Thoracolumbar junction sagittal Cobb angle (TLJS; the angle between the upper endplate of the T10 vertebra and the lower endplate of the L2 vertebra).
7. T1-spinopelvic inclination (T1-SPI; the angle between the vertical line and the line joining the center of the T1 vertebra and the axis of the femoral heads; this angle is positive when the hip axis lies in front of the T1 vertebral center) (17).
8. T9-spinopelvic inclination (T9-SPI; the angle between the vertical line and the line joining the center of the T9 vertebra and the axis of the femoral heads; this angle is positive when the hip axis lies in front of the T9 vertebral center) (17).

For all sagittal alignments, the angle is considered to be negative if the curve is lordotic and positive if the curve is kyphotic. The measurements were made with Surgimap spine medical imaging analysis software (Depuy Spine, Inc., USA).

### Statistical Analysis

All data are expressed as the mean ± standard deviation and were analyzed using SPSS software (version 18, SPSS Inc., Chicago, IL, USA). Independent sample *t* test analysis was performed to compare age, Cobb angle, Nash-Moe grade, traditional sagittal alignments (TK, TLJS, LL) and spinopelvic parameters (SS, PT, PI, T1-SPI, T9-SPI) between the two groups (kyphotic group and non-kyphotic group). Bivariate correlation tests were performed to determine whether there was a correlation between Nash-Moe grade, traditional sagittal alignments and spinopelvic parameters in thoracolumbar/lumbar AIS. Spearman correlation coefficients were determined to assess associations between ordinal variables, such as Nash-Moe grades. Pearson correlation coefficients were determined to assess associations between continuous variables. Stepwise multiple linear correlation analysis was employed to assess the following variables for independent correlation with TLJS: Nash-Moe grade, PI, T9-SPI, TK, and LL. The threshold for inclusion of individual variables in the multiple linear correlation model was *p* < 0.10. A *P* value < 0.05 was considered to be statistically significant.

## Results

There were 114 AIS patients (104 girls and 10 boys) included in the study. For all the patients, the mean age was 14.5 ± 1.5 years old (range, 11–18 y), and the Cobb angle of the main curves in these patients ranged from 40° to 60°, with an average of 45.9° ± 5.3° (Table 1). All patients were classified as having thoracolumbar/lumbar AIS.

**Table 1:** Sagittal and pelvic radiographic parameters.

**Parameter**	**All patients**	**Kyphotic Group**	**Non-kyphotic Group**	***t***	***P***
Number (%) of patients	114 (100)	44 (38.6)	70 (61.4)	–	–
Age (yr)	14.5 ± 1.5	14.8 ± 1.7	14.4 ± 1.5	1.081	NS
Primary Cobb angle (°)	45.9 ± 5.3	47.1 ± 5.6	45.1 ± 5.1	1.579	NS
Nash-Moe grade of AVR	2.3 ± 0.6	2.7 ± 0.6	2.1 ± 0.4	4.926	0.000^*^
Thoracic kyphosis (°)	15.9 ± 7.6	18.8 ± 7.4	14.0 ± 7.2	3.032	0.003^*^
TLJS (°)	7.1 ± 8.3	15.4 ± 3.7	2.0 ± 5.8	9.714	0.000^*^
Lumbar lordosis (°)	−48.7 ± 10.1	−46.1 ± 11.8	−50.4 ± 8.6	−1.765	NS
Sacral slope (°)	37.3 ± 8.3	32.9 ± 8.3	40.0 ± 7.1	−3.956	0.000^*^
Pelvic tilt (°)	4.6 ± 7.0	4.6 ± 6.5	4.6 ± 7.4	−0.021	NS
Pelvic incidence (°)	41.9 ± 9.8	37.6 ± 10.1	44.5 ± 8.8	−3.203	0.002^*^
T1-spinopelvic inclination (°)	5.1 ± 2.6	5.4 ± 2.8	4.9 ± 2.4	0.822	NS
T9-spinopelvic inclination (°)	7.4 ± 3.7	9.4 ± 3.7	6.2 ± 3.2	4.045	0.000^*^

*Values are the mean ± SD; SD, standard deviation; TLJS, thoracolumbar junction sagittal Cobb angle; AVR, apical vertebral rotation; NS, not significant (P ≥.05)*.

* *Statistically significant (P < 0.05)*.

The results of the independent samples *t* test analysis between the traditional sagittal alignments (TK, TLJS, and LL) and spinopelvic parameters (SS, PT, PI, T1-SPI, and T9-SPI) for the two groups are shown in Table 1. The patients in the non-kyphotic group had, on average, lower TK than patients in the kyphotic group (14.0 ± 7.2° vs. 18.8 ± 7.4°, respectively). However, the two groups had similar LL. For the spinopelvic parameters, the average SS and PI were obviously greater in the non-kyphotic group than in the kyphotic group (SS: 40.0 ± 7.1° vs. 32.9 ± 8.3°, respectively; PI: 44.5 ± 8.8° vs. 37.6 ± 10.1°, respectively). In contrast, the mean T9-SPI was significantly smaller in the non-kyphotic group than in the kyphotic group (6.2 ± 3.2° vs. 9.4 ± 3.7°, respectively). In addition, there were no significant differences in PT and T1-SPI between these two groups. For the Nash-Moe grade measurements of AVR, the mean grade in the kyphotic group was higher than that in the kyphotic group (2.7 ± 0.6 vs. 2.1 ± 0.4, respectively).

The correlation between the sagittal parameters and the spinopelvic parameters for all thoracolumbar/lumbar AIS patients is shown in Table 2. The SS and PI were found to be inversely associated with TLJS (SS: *r* = −0.474, *p* < 0.001; PI: *r* = −0.309, *p* = 0.008, respectively). However, T9-SPI and Nash-Moe grade were positively related to TLJS (T9-SPI: *r* = 0.457, *p* < 0.001; Nash-Moe grade: *r* = 0.506, *p* < 0.001, respectively). A moderate correlation was appreciated between TLJS and both TK and LL (TK: *r* = 0.291, *p* = 0.011; LL: *r* = −0.249, *p* = 0.031, respectively). There was no significant association between TLJS and PT or T1-SPI.

**Table 2:** Correlation coefficients (*r*) between the radiographic parameters.

**Parameters**	**Correlation coefficients (*r*)**	***P* value**
TK-T9-SPI^†^	0.463	<0.001^***^
TLJS-TK^†^	0.291	0.011^*^
TLJS-LL^†^	−0.249	0.031^*^
TLJS-SS^†^	−0.474	<0.001^***^
TLJS-PT^†^	0.128	0.272
TLJS-PI^†^	−0.309	0.008^**^
TLJS-T1-SPI^†^	0.158	0.176
TLJS-T9-SPI^†^	0.457	<0.001^***^
LL-SS^†^	0.780	<0.001^***^
LL-PT^†^	−0.188	0.106
LL-PI^†^	0.536	<0.001^***^
TLJS-Nash-Moe grade^†^^†^	0.506	<0.001^***^

*TLJS, thoracolumbar junction sagittal Cobb angle; TK, thoracic kyphosis; LL, lumbar lordosis; PI, pelvic incidence; SS, sacral slope; PT, pelvic tilt; T1-SPI, T1-spinopelvic inclination; T9-SPI, T9-spinopelvic inclination*.

*The correlation coefficients were calculated using Pearson's correlation analysis*.

*The correlation coefficients were calculated using Spearman's correlation analysis*.

* *Statistically significant (P < 0.05)*.

** *Statistically significant (P < 0.01)*.

*** *Statistically significant (P < 0.001)*.

Due to the geometrical relationship between the pelvic angles and sacral slope (PI = SS + PT), PI was a fixed anatomical parameter. When the “stepwise method” multiple linear regression analysis was conducted, PI was selected as the representative pelvic parameter. SPSS software automatically selected the most important independent variable/variables that contributed to TLJS variation, and only the Nash-Moe grade, T9-SPI and PI were entered. The equation was as follows, and the adjusted R^2^ was 0.410 (Table 3):

$$\begin{array}{l} \text{TLJS}=-2.322+5.585\times \text{Nash}-\text{Moegrade}+0.687\times \\ \text{ T}9-\text{SPI}-0.208\times PI. \end{array}$$

**Table 3:** Multivariate linear regression analysis of variables influencing the thoracolumbar junction sagittal Cobb angle.

**Variate**	**Thoracolumbar junction sagittal Cobb angle**			
	**Partial regression coefficient (B)**	**Standardized partial regression coefficient**	***t***	***P***
Nash-Moe grade of AVR	5.585	1.420	3.933	0.000^***^
T9-SPI	0.687	0.202	3.391	0.001^**^
Pelvic incidence (°)	−0.208	0.078	−2.684	0.009^**^

*AVR, apical vertebral rotation*.

** *Statistically significant (P < 0.01)*.

*** *Statistically significant (P < 0.001)*.

## Discussion

The relationship between the morphology of the pelvic vertebra and sagittal alignment of the spine has been definitively demonstrated not only in adults but also in adolescent populations (8, 18, 19). Since Duval-Beaupere et al. (20) emphasized the important effect of pelvic morphology in regulating an adequate sagittal balance, many studies have illustrated the relationship between the morphology of pelvic vertebra and spinal balance both in normal and pathologic conditions (17, 18, 21, 22). It has been reported in the literature that the thoracolumbar junction should be at least straight and preferentially slightly lordotic in the sagittal plane (14). It has also been mentioned that hyperkyphotic TLJS plays an important role in the development of symptomatic sagittal imbalance (14). Furthermore, a slight or moderate kyphotic thoracolumbar junction can be observed in AIS, especially in both double major and thoracolumbar/lumbar AIS (8, 9, 12). To date, although TLJS has been demonstrated to be positively correlated with a greater axial vertebral rotation of the scoliotic spine (13), no study examining the correlation between TLJS and spinopelvic morphology in AIS patients has been published. The purposes of this current study were to ascertain the correlation between spinopelvic alignment and TLJS in thoracolumbar/lumbar AIS and, more importantly, to explore the potential predictive factors for hyperkyphotic TLJS in the sagittal plane.

In the present study, by evaluating standard lateral radiography, 44 patients (38.6% of all patients) with thoracolumbar/lumbar scoliosis showed 15.4 ± 3.7° TLJS in the sagittal plane. However, this result was quite different from Ni's study (13) because of the definition of thoracolumbar junction kyphosis. Ni et al. (13) measured the thoracolumbar junctional sagittal curve from T11 to L2; in contrast, our definition of this curve is from T10 to L2. Regarding the Nash-Moe grade measurement of AVR, the mean grade in the kyphotic group was higher than that in the non-kyphotic group, which was comparable to a previous study (13).

As previously reported in the literature (21), a geometric construction revealed that there is a geometrical relationship between the pelvic angles and sacral slope such that pelvic incidence is equal to the algebraic sum of the angles of sacral slope and pelvic tilt (PI = SS + PT). The PI, as shown in previous studies, was significantly greater in AIS patients than in normal adolescents without scoliosis (8, 9). However, the PI in AIS patients was similar to that of normal adolescents according to Yong's study (10). Additionally, PI could influence the ability of the spine to compensate for its deformity (23) by tilting the pelvis backwards, which would decrease the SS. In the current study, a significantly smaller PI was found in the kyphotic group (37.6 ± 10.1°) than in the non-kyphotic group (44.5 ± 8.8°); otherwise, the PT was not different between these two groups. By the same token, thoracolumbar/lumbar AIS patients with smaller PI were more likely to have kyphotic TLJS in the sagittal plane; the patients with a smaller PI were not able to easily compensate for the kyphosis deformity by reducing the SS or inducing the PT because the ability of the individual to vary the SS or PT to compensate for sagittal imbalance depends on the size of PI (23). Therefore, the mean SS in the kyphotic group (32.9 ± 8.3°) was comparably smaller. These findings strongly support the theory of the sagittal compensatory mechanism previously proposed by Roussouly (23): patients with a small PI do not have adequate capacity to induce the amount of PT required to restore balance. However, the mean value of the PI (41.5 ± 9.7°) in all patients in our study was much smaller than the mean values in most published studies (8, 9, 17), which was similar to that of Yong's study (10); this may possibly be due to differences in the spine between Asian and Caucasians. That is, PI value may vary with ethnicity (10).

The T1-SPI and T9-SPI have been used for assessment of the global balance and truncal inclination of the sagittal spine (24). The advantage of these two angular parameters helps avoid the error inherent in measuring offsets even in noncalibrated radiographs (24). The mean T1-SPI angle was similar in these two groups, which indicated that patients with thoracolumbar/lumbar scoliosis in the coronal plane had good global trunk balance in the sagittal plane. The T9 vertebra had been shown to have a rather fixed location regarding the gravity line according to a previous study (25). However, in the present study, the T9-SPI angle in the kyphotic group was significantly greater than that in the non-kyphotic group, which means that the vertebra in the thoracolumbar junction (from T10 to L2) was much further from the gravity line. This result also verified the presence of hyperkyphosis in the thoracolumbar junction region in thoracolumbar/lumbar AIS patients from the other side.

Previous studies have shown varying results on normal values of acceptable TK in the spine, ranging from 20° to 40° (26), although reference values of 20° to 50° have also been suggested (16, 27). The mean value of TK in all patients (15.6 ± 7.5°) was below the normal range reported in the literature and has also been demonstrated in a previous study (13). However, Upasani et al. (9) reported that there was no significant difference in the TK between patients with thoracolumbar/lumbar scoliosis and normal control subjects. The differences between those studies may, in part, be attributed to the different population samples. The present results showed that the mean value of TK was significantly greater in the kyphotic group (18.9 ± 7.4°) than in the non-kyphotic group (13.4 ± 6.8°), possibly due to the location of TK (T5–T12). In other words, the upper vertebrae (T10, T11, and T12) in the thoracolumbar junction region were the lower thoracic vertebrae of the spine. Therefore, the upper curve of the thoracolumbar junction in the sagittal plane influenced the lower TK curve.

Pelvic morphology has been shown to influence sagittal spinal alignment and balance (9, 28, 29). In the present study, a strong positive correlation was observed between LL and SS (*r* = 0.780, *P* < 0.001) and between LL and PI (*r* = 0.536, *p* < 0.001) in all patients. These results were consistent with the relationship between PI and lumbar sagittal alignment described in previous studies, which was crucial for balancing the upright spine not only in normal spines but also in scoliotic spines (8, 20, 30, 31). In addition, LL, SS, and PI were inversely associated with TLJS (LL: *r* = −0.249, *p* = 0.031; SS: *r* = −0.474, *p* < 0.001; PI: *r* = −0.309, *p* = 0.008, respectively) when the bivariate correlation analysis was conducted. In contrast, TK, T9-SPI, and Nash-Moe grade were positively correlated with TLJS (TK: *r* = 0291, *p* = 0.011; T9-SPI: *r* = 0.457, *p* < 0.001; Nash-Moe grade: *r* = 0.506, *p* < 0.001, respectively). Furthermore, the “stepwise method” multiple linear regression analysis showed that the most important independent variables that contributed to TLJS were Nash-Moe grade, T9-SPI, and PI. The equation was as follows, and the adjusted R^2^ was 0.410 (implying that 41.0% of TLJS variation could be accounted for by Nash-Moe grade, T9-SPI, and PI):

$$\begin{array}{l} \text{TLJS}=-2.322+5.585\times \text{Nash}-\text{Moegrade}+0.687\times \\ \text{ T}9-\text{SPI}-0.208\times \text{ PI}. \end{array}$$

TLJS did show an increasing trend with increasing Nash-Moe grade and T9-SPI or decreasing PI. That is, among adolescents with idiopathic scoliosis and a thoracolumbar/lumbar curve pattern, a higher Nash-Moe grade indicated that more serious thoracolumbar junction kyphosis would occur, which agrees with Ni's study (13). Nash-Moe grade had the highest predictive value and impact on TLJS, signified by the high beta and *t* values. More importantly, PI and T9-SPI can both significantly impact TLJS, and these two variables as predictive factors for hyperkyphotic TLJS have not yet been described. Spine surgeons should pay more attention to the degree of AVR, T9-SPI, and PI when dealing with thoracolumbar/lumbar scoliosis with thoracolumbar junction kyphosis, such as in the context of bracing therapy in the outpatient department and with strategies for surgical correction of scoliosis by restoring the thoracolumbar junction region at least straight and preferentially slightly lordotic.

Several limitations should be mentioned in the current study. First, the standard long-cassette standing upright AP and lateral radiographs may not have demonstrated the “true” deformity of scoliosis in the 3D planes. In addition, the magnitude of the thoracolumbar junction sagittal Cobb angle may be overestimated, especially for those with serious wedging of vertebra or greater axial rotation of the spine. More advanced techniques should be used to estimate the “true” deformity of the spine, such as those using the EOS three-dimensional reconstruction imaging system (32). Second, there was only one type of AIS included in this study.

It was demonstrated that among patients with thoracolumbar/lumbar scoliosis, the thoracolumbar junction sagittal Cobb angle (TLJS) was positively correlated with greater AVR in the coronal plane and greater T9-SPI in the sagittal plane and inversely associated with PI. Spine surgeons should pay more attention to the degree of AVR, T9-SPI, and PI when dealing with thoracolumbar/lumbar scoliosis with thoracolumbar junction kyphosis, such as in the context of bracing therapy in the outpatient department and with strategies for surgical correction of scoliosis by restoring the thoracolumbar junction region at least straight and preferentially slightly lordotic.

## Data Availability Statement

The original contributions presented in the study are included in the article/supplementary material, further inquiries can be directed to the corresponding author/s.

## Ethics Statement

The studies involving human participants were reviewed and approved by the ethics review board of the Affiliated Zhangjiagang Hospital of Soochow University. Written informed consent to participate in this study was provided by the participants' legal guardian/next of kin. Written informed consent was obtained from the individual(s), and minor(s)' legal guardian/next of kin, for the publication of any potentially identifiable images or data included in this article.

## Author Contributions

XL and JZ conceived the study and design, drafted the main manuscript text. FY and LW analyzed and interpreted the data. WS and YQ performed critical revision of the manuscript. All authors contributed to the article and approved the submitted version.

## Conflict of Interest

The authors declare that the research was conducted in the absence of any commercial or financial relationships that could be construed as a potential conflict of interest.
